# Comprehensive Approach for Rehabilitation of a Completely Edentulous Patient: A Case Report

**DOI:** 10.7759/cureus.68312

**Published:** 2024-08-31

**Authors:** Shruti Deshmukh, Sweta G Pisulkar, Akansha Bansod, Arushi Beri, Ritul Jain

**Affiliations:** 1 Department of Prosthodontics and Crown & Bridge, Sharad Pawar Dental College and Hospital, Wardha, IND; 2 Department of Prosthodontics and Crown & Bridge, Sharad Pawar Dental College And Hospital, Wardha, IND

**Keywords:** cu-sil denture, neutral zone, flabby tissue, complete denture, impression techniques

## Abstract

Complete edentulism poses significant challenges to oral function, esthetics, and overall quality of life. The use of complete dentures remains a cornerstone in rehabilitating such patients and restoring masticatory function, phonetics, and facial esthetics. However, to improve patients’ function, it is important to restore the stomatognathic system.

The rehabilitation process begins with a thorough assessment of the patient’s oral health status, including evaluation of the remaining supporting structures and the surrounding soft tissues. Impressions and jaw relation records are meticulously obtained to ensure accurate denture fabrication. Proper denture retention, stability, and occlusion are crucial for successful rehabilitation. Beyond functional aspects, attention to esthetics plays a pivotal role in patient satisfaction and acceptance of complete dentures. Customization of denture teeth in terms of shape, shade, and arrangement is essential to achieve natural-looking smiles and facial harmony.

Complete dentures remain a valuable treatment modality for the comprehensive rehabilitation of completely edentulous patients. Through a combination of meticulous treatment planning, advanced techniques, and patient-centered care, we can achieve successful outcomes in restoring oral function and thus improving the overall quality of life for such patients. Thus, this case report outlines a simple, economical, and useful rehabilitation plan for a patient with an edentulous maxillary and mandibular arch with flabby tissue and a resorbed mandibular ridge.

## Introduction

The effectiveness of a complete denture often depends on its support and retention. To ensure optimal retention, stability, and support during use, it’s crucial that the final impression of a denture accurately captures the complete denture-bearing area [1]. However, challenges arise when the quality of these areas is inadequate, such as the presence of flabby ridges or severely resorbed ridges, particularly in the mandible, where resorption is often more pronounced than in the maxilla [2,3]. These issues can result in unstable denture positioning and dissatisfaction for the wearer while also compromising the quality of the denture-bearing tissue. Therefore, proper prosthodontic management is essential to restoring the patient’s function, esthetics, and speech [4].

This case report dwells on the management of flabby tissue with the Zafarullah and Hobkirk combination impression technique, followed by the recording of the neutral zone for severely resorbed mandibular ridges [5]. The goal of the neutral zone technique is to create a denture that blends in with the surrounding oral structures and is sculpted by muscle function. Teeth are positioned using this approach so that they remain in a safe, protected zone and that the forces applied by the tongue and cheek muscles are neutralized [6].

Additionally, a Cu-sil denture was made for the maxillary arch. Cu-sil dentures are designed to safeguard the alveolar process and preserve the remaining natural teeth, which will ultimately improve denture stability and retention [7].

## Case presentation

A 65-year-old male patient reported to the Department of Prosthodontics with the chief complaint of a loose denture and difficulty speaking and eating with it. He has been a complete denture wearer for the past six years, and due to the inconvenience of using his previous denture, he wanted to replace it.

**Figure 1:**
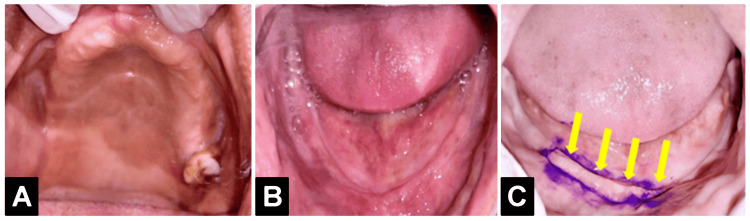
Intraoral images: (A) maxillary arch; (B) mandibular arch; (C) flabby tissue

The treatment plan for this patient was to construct an upper Cu-sil denture with a lower complete denture using the neutral zone technique. Figure 1 shows intraoral preoperative images.

On the first visit, preliminary impressions were made for both the maxillary and mandibular arches with impression compound (type 1 Y-dents, MDM Corporation, Delhi, India). The impression was immediately poured into dental plaster, and the primary cast was obtained from this.

**Figure 2:**
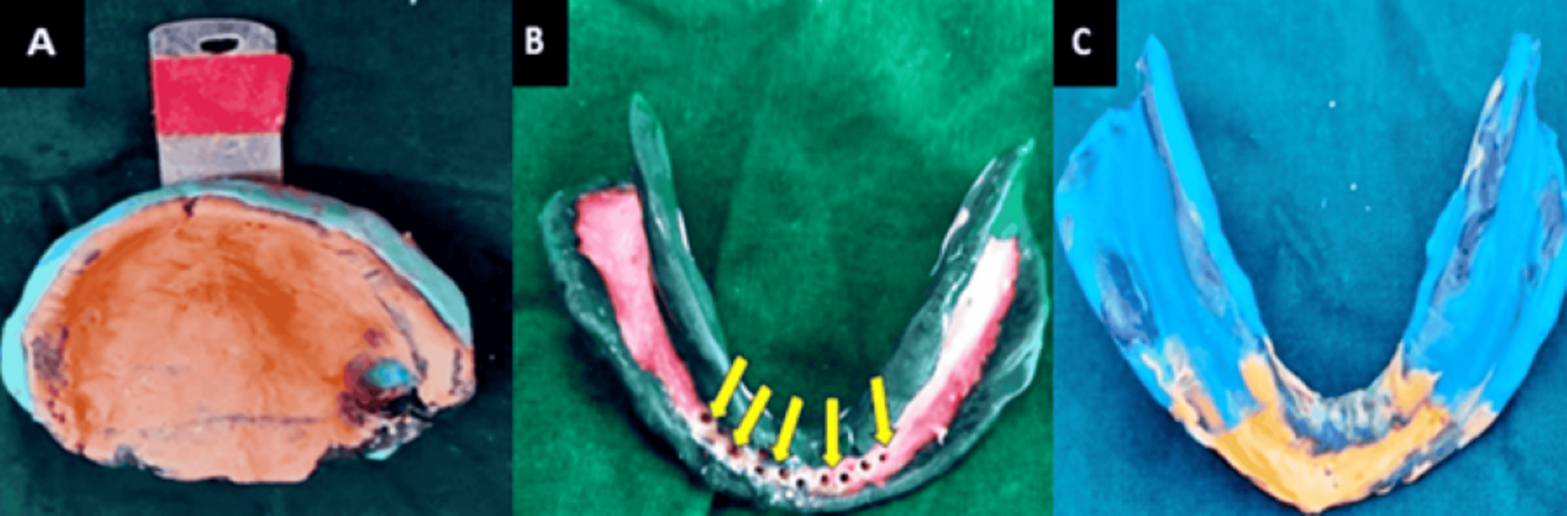
(A) Maxillary pickup impression; (B) mandibular border with bolding perforations made in the tray in flabby tissue region; (C) mandibular final impression Arrows depict perforations made in the region of flabby tissue.

On the second visit, a custom tray was fabricated on the primary cast for both maxillary and mandibular casts, on which the border molding was done using a low-fusing green stick compound. During the final impression, a pick-up impression was made for the maxillary arch using zinc oxide eugenol (DPI impression paste Bombay Burmah Trading Corporation Ltd., Mumbai, India) and alginate (Zhermack hydrogum 5, Italy). For the mandibular arch, followed by border molding, the final impression was made with a medium body. In the area of flabby tissue, perforation was created to keep the flabby tissue static, and an impression was made for the anterior region using the light body. Figure 2 shows border molding and the final impression for the maxillary arch and the Zafarullah and Hobkirk combination impression technique for the mandibular arch.

On the third visit, on the master cast, the temporary denture bases were fabricated using a cold‑curing acrylic material (Bombay Burmah Trading Corporation Ltd.). After which, the wax rim was fabricated on record bases. These wax rims were used to record jaw relation. First, the initial vertical dimension was recorded, and centric relation records were made. Two conspicuous areas on the patient’s face, one on the nose and another on the chin, were marked while the patient was made to sit in a straight, upright position. Between these two positions, the vertical dimension at rest was measured using a divider and a 12-inch ruler. The vertical dimension at occlusion was 69 mm, and the determined vertical dimension at rest was 72 mm.

**Figure 3:**
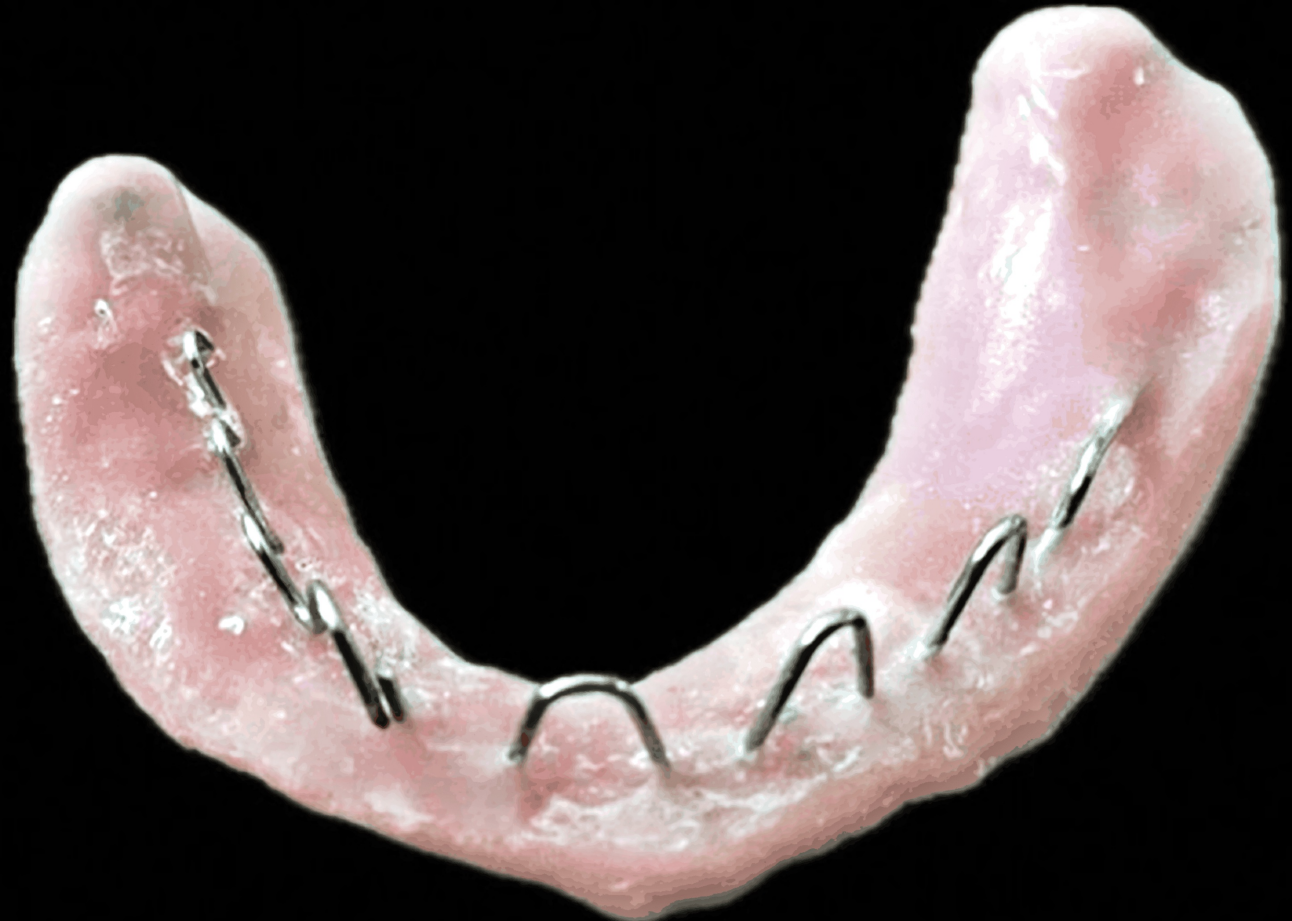
Modified record base with spurs made on it that will hold the material during neutral zone recording

The mandibular record base was removed, and a modified record base was made with spurs that would hold the impression material during recording. Figure 3 shows a modified record base with spurs made of orthodontic wire.

**Figure 4:**
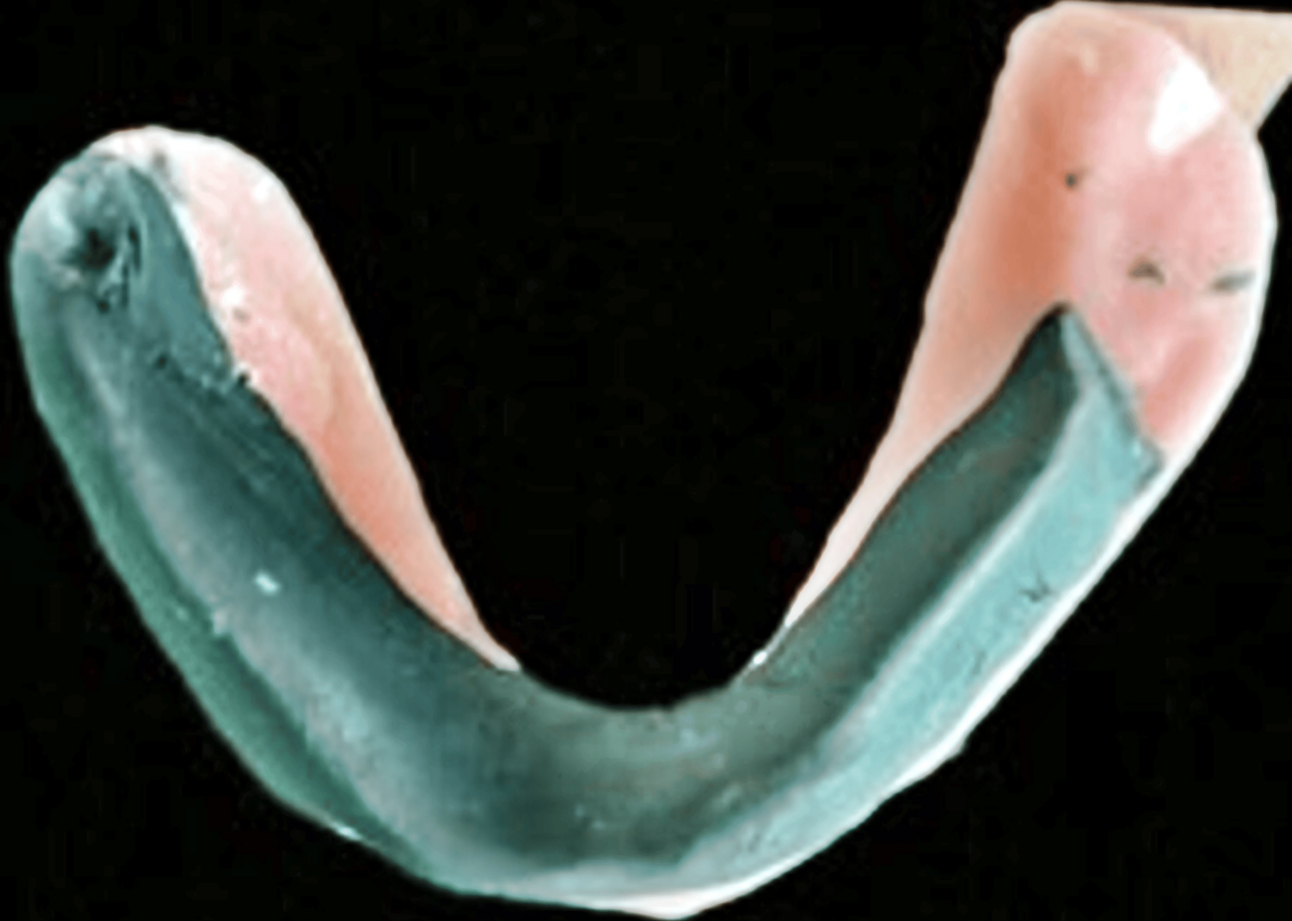
Neutral zone recorded using Admix

Next, a mixture of impression material and green stick molding material in a ratio of 3:7 was mixed on the modified mandibular denture base and placed at the desired height. Patients were then asked to perform movements such as swallowing and pronouncing vowels, such as aa, ee, oo, and other routine mandibular movements, which included swallowing, sucking of the lips, and tongue movements that aided in recording the neutral zone area (Figure 4).

**Figure 5:**
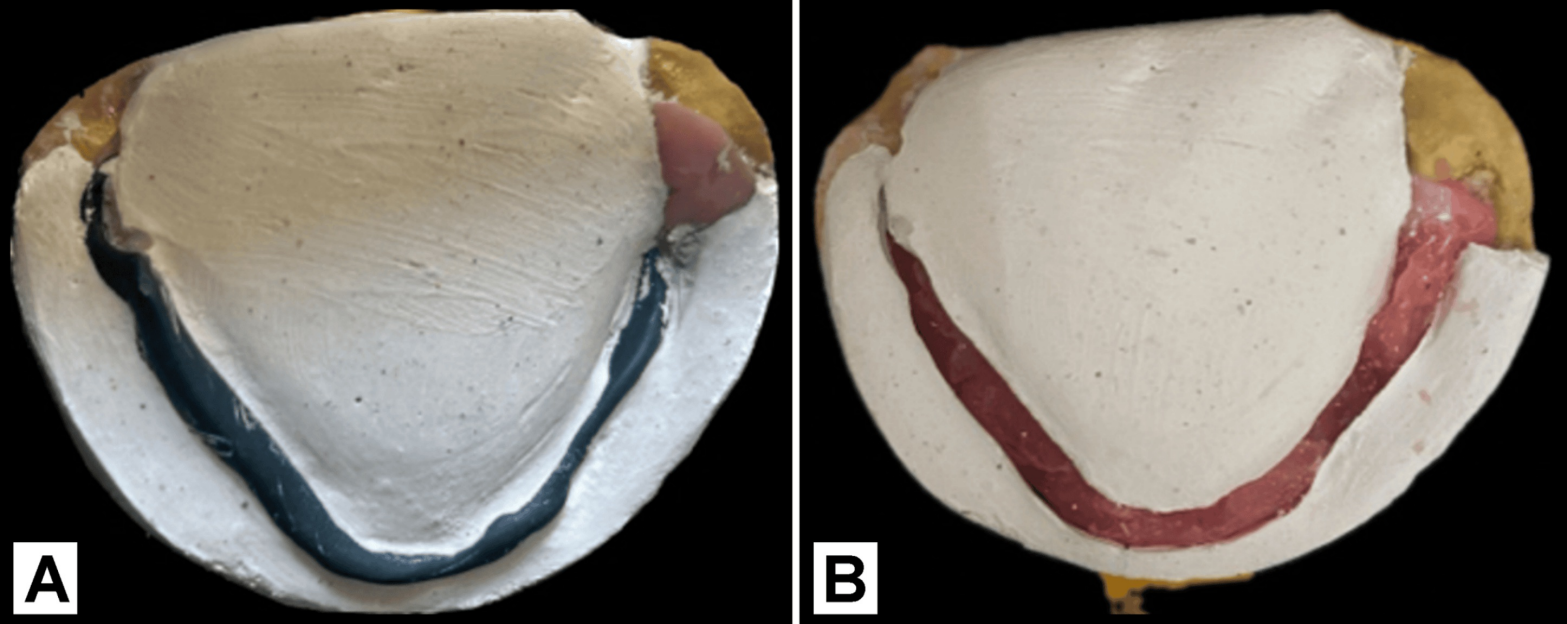
(A) Plaster index records over the neutral zone; (B) molten modeling wax poured into the space between the index

Next, the mandibular temporary denture base with the neutral zone recorded is removed. The neutral zone is held in place with a plaster grid. Melted modeling wax is poured between the marks, and the wax flows within the neutral zone to form the shape of the rim. Figure 5 shows plaster index records over the neutral zone, followed by wax poured into the space created by the plaster index.

On the fourth visit, to assess intraoral occlusion, esthetics, and record base stability, a wax try-in was conducted. Following that, the denture was processed with heat-cured acrylic resin. After that, the denture was finished and polished. Prior to denture placement, the mandibular denture was then checked once more using the plaster index.

**Figure 6:**
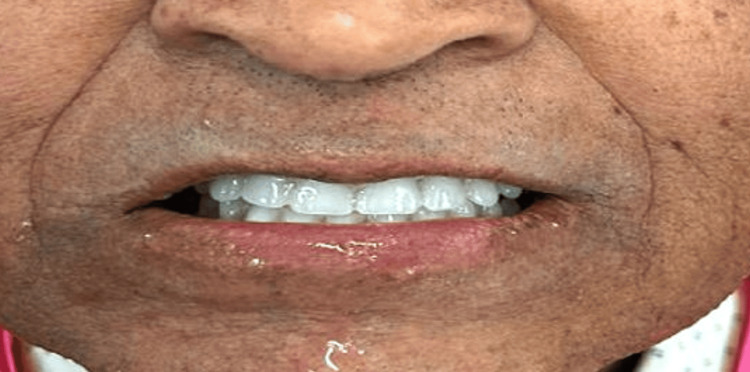
Post-operative photograph of the patient after denture insertion

In the final visit, the denture was delivered to the patient. The denture was checked for retention, stability, and occlusion. The patient was made comfortable with the denture. Post-denture instructions were given to the patient, and periodic follow-up appointments were scheduled for the patient. Figure 6 shows a postoperative image of the patient.

## Discussion

In an atrophic mandible, the major concern is the inability of the ridge and the overlying tissues to bear the masticatory loads. This occurs due to the muscle attachments, which gradually become closer to the ridge due to the resorption process. These muscles apply a dislodging force onto the denture prosthesis upon mastication [8]. Flabby tissue and severely resorbed mandibular ridges are the problems dentists encounter during the fabrication of a complete denture. Hence, meticulous treatment planning is a must in such cases, which starts with the first step of impression-making. It can be done mainly through three approaches.

Surgical approach

Following surgical removal of the loose tissues, a poor ridge is preferable to none at all. The benefit of the surgical method is that it creates a stable denture-bearing surface, improving the prosthesis’s stability. One of its drawbacks is the possibility of a reduction in vestibular height, necessitating a second vestibuloplasty procedure. Patients who are unwilling to have surgery should not have it (Pai et al., 2014) [9].

Implant prosthesis

It draws its support from the underlying bone; therefore, little or no tissue area support is required. The implant-supported prosthesis has limitations with regard to patient economics and operation duration. Additional elements need to be taken into account [10].

Conventional prosthetic management

Several different conventional techniques are given in the literature for flabby tissue management, such as the window impression technique (minimally displacive impression technique). In this method, after a custom tray window is fabricated in an area where flabby tissue is present, then border molding is done. After that, the final impression is made, and in that area where the window is created, the impression is made with dental plaster, but the main drawback in this case is distortion of dental plaster [11]. Another method is the selective pressure impression technique, in which additional relief is provided in the area of flabby tissue, and then the custom tray is made over the spacer, the area where the double spacer is applied. In that area, trays are made with clear acrylic and the final impression with monophase polyvinyl siloxane material (Pai et al., 2014) [9].

Selective perforation tray technique

In this case, the flabby tissue is recorded with light body PVS, the normal tissue is recorded with ZOE, and the peripheral border and PPS area are recorded with impression compound [12,13].

In this case report, the Zafarullah and Hobkirk combination impression technique is used, which is a modification of Watson’s window impression technique. In order to accurately record the tissue under rest position, Zafarullah and Hobkirk changed the approach, wherein a custom tray with a window or opening is produced over the flabby tissues (typically anterior), and the custom tray over the flabby tissue area is then perforated.

Additionally, the use of the improvised neutral zone technique increases stability and control of the lower denture (by reducing displacement pressures) for patients with weak neuromuscular coordination, hence providing an advantage of facilitation of muscle control.

## Conclusions

A prosthodontist provides patients with atrophic ridges and functional and esthetic dental treatments, which is an invaluable service. Hence, a conservative approach is needed for the complete rehabilitation of severely compromised alveolar ridges. Patients with atrophic ridges and flabby tissue primarily complain of pain and looseness in their mandibular dentures, and the methods explained in the case report can benefit such patients. It has been demonstrated that this method of recording neutral zones and using an accurate impression technique helps people who are dissatisfied with their mandibular dentures. Furthermore, in a patient with inadequate muscular coordination, the admix substance helped record the functions of the mouth musculature. The effectiveness of the therapy depends on the prosthesis’s capacity to tolerate the different forces operating on it and the remaining ridge region tissues, which work in tandem with a well-made prosthesis to oppose these displacement forces. In order to provide patients with a complete denture prosthesis that is functional, physiological, and psychologically acceptable, the clinician should be aware of the benefits of each technique and apply them in clinical practice.
